# Serum levels of miR-126 and miR-223 and outcomes in chronic kidney disease patients

**DOI:** 10.1038/s41598-019-41101-8

**Published:** 2019-03-14

**Authors:** Ophélie Fourdinier, Eva Schepers, Valérie Metzinger-Le Meuth, Griet Glorieux, Sophie Liabeuf, Francis Verbeke, Raymond Vanholder, Benjamin Brigant, Anneleen Pletinck, Momar Diouf, Stéphane Burtey, Gabriel Choukroun, Ziad A. Massy, Laurent Metzinger, on behalf of the European Uremic Toxin Work Group-EUTox, Angel Argiles, Joachim Beige, Philippe Brunet, Gerald Cohen, Omar Abou Deif, Pieter Evenepoel, Danilo Fliser, Ivo Fridolin, Andreas Gmerek, Joachim Jankowski, Vera Jankowski, Roos Masereeuw, Harald Mischak, Alberto Ortiz, Alessandra Perna, Juan Mariano Rodriguez-Portillo, Joost Schanstra, Goce Spasovski, Dimitrios Stamatialis, Sonja Steppan, Markus Storr, Bernd G. Stegmayr, Peter Stenvinkel, Paul J. Thornalley, Andrej Wiecek

**Affiliations:** 1 INSERM U1088, CURS, Université de Picardie Jules Verne, Amiens, France; 2 Nephrology Dialysis and Transplantation Department, Amiens University Hospital, Amiens, France; 3 Nephrology Section, Department of Internal Medicine and pediatrics, Ghent University Hospital, Corneel Heymanslaan 10, 9000 Ghent, Belgium; 4 INSERM U1148, Laboratory for Vascular Translational Science (LVTS), UFR SMBH, Université Paris 13-Sorbonne Paris Cité, 93017 Bobigny Cedex, France; 5 Pharmacology Department and Clinical Research Department, Amiens university hospital, Amiens, France; 6 HEMATIM EA4666, CURS, Université de Picardie Jules Verne, CHU Amiens Sud, Avenue René Laënnec, Salouel, F-80054 Amiens, France; 7 Biostatistics Unit, Clinical Research and Innovation Department, Amiens-Picardie University Hospital, F-80054 Amiens, France; 8 Aix Marseille University, INSERM, INRA, C2VN Marseille, France; 9 Ambroise Paré Hospital, Division of Nephrology, APHP, Paris Ile de France Ouest (UVSQ) University, et INSERM 1018 Eq. 5, CESP, Boulogne Billancourt et Villejuif, Paris, France; 10 EA7288, Université de Montpellier, Montpellier, France; 11 Leiter KfH-Nierenzentrum und Klinikum St. Georg, Leipzig, Germany; 12 Medizinische Universität Wien Univ. Klinik für Innere Med, Medizinische Universittät Wien, Wien, Austria; 13 Borsteler Chaussee 17-21, DE-22459 Hamburg, Germany; 14 Laboratory of Nephrology Department of Immunology and Microbiology, Leuven, Belgium; 15 Klinik für Innere Medizin, Universitätsklinikum des Saarlandes, Homburg, Germany; 16 Technomedicum, Tallinn University of Technology, Tallinn, Estonia; 17 Braun Avitum AG Medical Scientific Affairs, Melsungen, Germany; 18 Institute of Cardiovascular Research, Aachen University, Aachen, Germany; 19 Utrecht Institute for Pharmaceutical Sciences, Utrecht, The Netherlands; 20 LAB MOSAIQUES DIAGNOSTICS & THERAPEUTICS, Hannover, Germany; 21 Department of Nephrology IIS - Reyes Católicos, Madrid, Spain; 22 Seconda Università degli Studi di Napoli, Facoltá di Medicina Napoli, Napoli, Italy; 23 University Hospital Reina Sofia Unidad de Investigacion Avda Menendez, Cordoba, Spain; 24 INSERM, U1048 Toulouse, France; 25 Department of Nephrology University Clinical Center, Skopje, Macedonia; 26 University of Twente; Faculty of Science and Technology, Twente, The Netherlands; 27 Fresenius Medical Care Deutschland GmbH, Bad Homburg, Germany; 28 Baxter International R&D, Hechingen, Germany; 29 Div. of Nephrology, Univ. Hospital, Umea, Sweden; 30 Nephrology Department, Karolinska University Hospital, Stockholm, Sweden; 31 Warwick Medical School & Systems Biology Centre, University of Warwick, Coventry, United Kingdom; 32 Medical University of Silesian, Katowice, Poland

## Abstract

Several microRNAs (miRNAs) have been linked to chronic kidney disease (CKD) mortality, cardiovascular (CV) complications and kidney disease progression. However, their association with clinical outcomes remains poorly evaluated. We used real-time qPCR to measure serum levels of miR-126 and miR-223 in a large cohort of 601 CKD patients (CKD stage G1 to G5 patients or on renal replacement therapy – CKD G5D) from Ghent University Hospital and 31 healthy controls. All-cause mortality and cardiovascular and renal events were registered as endpoints over a 6 year follow-up period. miR-126 levels were significantly lower from CKD stage G2 on, compared to controls. The serum levels of miR-223 were significantly lower from CKD stage G3B on. When considering overall mortality, patients with levels of either miR-126 or miR-223 below the median had a lower survival rate. Similar results were observed for CV and renal events. The observed link between the two miRNAs’ seric levels and mortality, cardiovascular events or renal events in CKD appears to depend on eGFR. However, this does not preclude their potential role in the pathophysiology of CKD. In conclusion, CKD is associated with a decrease in circulating miR-223 and miR-126 levels.

## Introduction

Chronic kidney disease (CKD) is a major public health burden worldwide. This condition, and end-stage kidney disease in particular, is frequently associated with disorders of mineral and bone metabolism (CKD-MBD), and an increased cardiovascular risk in which progressive vascular calcification leads to a high cardiovascular mortality rate^1,2^. The identification of prognostic biomarkers is therefore of the utmost relevance to CKD patients.

MicroRNAs (miRNAs) are short, single-stranded, non-coding RNAs that downregulate gene expression through translational repression or degradation of messenger RNA (mRNA) by binding to the mRNA’s 3’-untranslated region^3^. miRNAs are highly conserved; the first miRNA was discovered in 1993 in *Caenorhabditis elegans* and the first human miRNAs were identified in 2000^4^. About 3000 miRNAs have now been identified in the human genome^3^. Their biogenesis and mode of action have been extensively reviewed elsewhere^5^.

Dysregulation of miRNAs has been linked to the pathophysiology of many diseases, including kidney and cardiovascular diseases^5–7^. Our team reported that miR-126 and -223 levels were altered in the aortic wall and serum in murine models of CKD^8^, and in the microcirculation of the brain of CKD mice^9^. Human targets of these miRNAs are shown in Supplementary Table 1.

miR-223 is considered to be a hematopoietic factor^10^ with a preponderant role in granulopoiesis^11,12^. It regulates cholesterol homeostasis and cardiac glucose metabolism, but it is also known to be an onco-miR^13–15^. In vascular disease, circulating miR-223 is able to penetrate the vascular smooth muscle cells (VSMCs) and act as an endocrine signal to regulate cell proliferation, migration and apoptosis^16,17^. *In vitro*, exposure of human VSMCs to pathological levels of inorganic phosphate (Pi) increases miR-223 expression as well as cell migration^18^. Finally, Ulbing *et al*. reported in a limited cohort of 140 patients, in CKD stages G3 to G5 or after kidney transplantation, that expression levels of miR-223-3p were lower in stage G4 and G5 CKD patients than in healthy controls^19^.

miR-126 is a pro-angiogenic miRNA present in endothelial cells^20^, and is highly expressed in vessels^21^. This miRNA has an important role in vascular dysfunction, since it enhances endothelial proliferation and endothelialization of large vessels, which in turn attenuates atherosclerosis. In a murine model of CKD, miR-126 is overexpressed in the aorta, along with the upregulation of *SDF-1*^8^, a chemokine protein playing an important role in angiogenesis recruiting endothelial progenitor cells from the bone marrow. During tissue repair, in a mouse model of atherosclerosis, endothelial cells release miR-126 in apoptotic bodies, inducing *SDF-1*-dependent vascular repair^22^. Furthermore, miR-126 overexpression in bone marrow cells has recently been shown to promote vascular integrity following kidney injury by contributing to the recovery of the kidney microvasculature^23^. Plasma levels of miR-126 are abnormally low in type 2 diabetes^24^, and low plasma levels are associated with a poor prognosis in renal cell carcinoma^25,26^.

Recent studies have shown that miRNAs in blood^27^ and other body fluids^28^ are remarkably stable. They are protected from endogenous RNase activity by their binding to Argonaut proteins or their uptake in exosomes^29^ or microvesicles^30^. They are also transported by nucleophosmin 1^31^, endothelial apoptotic bodies^22^ and high density lipoprotein (HDL)^32^. In view of the minimally invasive nature of blood and body fluid sampling and the stability of miRNAs, they may be useful as diagnostic and/or prognostic biomarkers.

The objectives of the present study were (i) to study the expression of circulating miR-126 and miR-223 in a cohort of 601 CKD patients and 31 healthy controls, and (ii) to evaluate the link between these miRNAs and cardiovascular and all-cause mortality. The secondary objective was to evaluate the association between the initial expression of miR-126 and miR-223 in serum and renal events and the decline of kidney function.

## Results

### Characteristics of the study population

**Figure 1:**
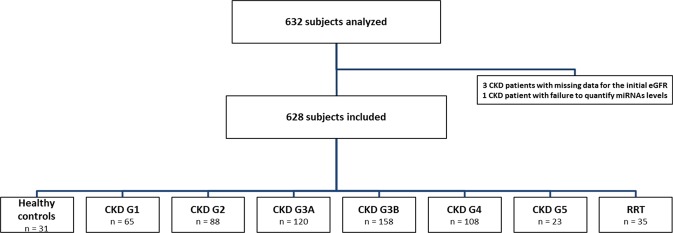
Study flow chart.

A total of 632 subjects, 601 CKD patients and 31 healthy controls were enrolled of which 628 were included in the final analysis (Fig. 1). Three CKD patients had missing data for the initial eGFR and, in one CKD patient, miRNA quantification in serum was impossible. The patients included in the analysis were divided into seven CKD groups, including patients on dialysis (22 patients on hemodialysis and 13 patients on peritoneal dialysis) and healthy controls. The clinical and biochemical characteristics of the study population are summarized in Table 1 and Supplementary Table 2.

**Table 1:** Baseline characteristics of the cohort participants overall and by group (CKD patients, patients on RRT, and healthy controls).

n	Cohort	Healthy	CKD G1	CKD G2	CKD G3A	CKD G3B	CKD G4	CKD G5	CKD G5D	P value*
	628	31	65	88	120	158	108	23	35	
Age, years	64 [49–74]	30 [25–58]^¥^	38 [28–50]	54 [43–62]	66 [56–73]	71 [64–78]	73 [63–81]	72 [57–78]	66 [62–75]	**p** **<** **0.05**
Male, n (%)	365 (58)	13 (42)	32 (49)	50 (57)	68 (57)	96 (61)	72 (67)	11 (48)	23 (66)	p = 0.26
Caucasian, n (%)	619 (99)	31 (100)	63 (97)	85 (97)	118 (98)	158 (100)	106 (98)	23 (100)	35 (100)	p = 0.36
BMI, kg/m²	27 [23–31]	22 [20–23]^¥^	25 [22–28]	27 [23–30]	27 [24–31]	29 [25–32]	28 [24–32]	26 [24–29]	26 [23–32]	**p** **<** **0.05**
Mean blood pressure, mmHg	97 [90–105]	93 [86–99]	95 [89–101]	99 [90–106]	96 [89–105]	98 [93–106]	97 [90–107]	99 [91–112]	92 [76–97]	**p** **<** **0.05**
Hypertension, n (%)	537 (86)	4 (13)^¥^	48 (73)	71 (81)	110 (92)	153 (97)	106 (98)	22 (96)	23 (66)	**p** **<** **0.05**
Diabetes, n (%)	182 (29)	0 (0)^¥^	9 (14)	21 (24)	28 (23)	65 (41)	42 (39)	9 (39)	8 (3)	**p** **<** **0.05**
Cardiovascular history, n (%)	53 (9)	0 (0)^¥^	1 (2)	4 (5)	6 (5)	20 (13)	18 (17)	2 (9)	2 (6)	**p** **<** **0.05**
**Etiology**, n (%)	n = 597									
Nephroangiosclerosis	105 (17.5)		1 (2)	10 (11)	26 (22)	31 (20)	29 (27)	4 (17)	4 (11)	
Diabetic nephropathy	93 (15.5)		4 (6)	9 (10)	11 (9)	42 (27)	20 (18)	2 (9)	5 (14)	
ADPKD/Alport	41 (7)		8 (12)	12 (14)	7 (6)	6 (4)	4 (4)	2 (9)	2 (6)	
Unique kidney	48 (8)		1 (2)	8 (9)	14 (12)	14 (9)	10 (9)	1 (4)	0 (0)	
Lupus/vasculitis	39 (6.5)		14 (21)	9 (10)	10 (8)	4 (2)	1 (1)	1 (4)	0 (0)	
Membranous nephropathy	8 (1)		0 (0)	4 (5)	2 (2)	1 (1)	1 (1)	0 (0)	0 (0)	
IgA nephropathy	27 (4.5)		9 (14)	4 (5)	4 (3)	4 (2)	5 (5)	1 (4)	0 (0)	
Urologic/reflux nephropathy	17 (3)		1 (2)	3 (3)	3 (3)	5 (3)	1 (1)	2 (9)	2 (6)	
Tubulo-interstitial nephritis	40 (7)		6 (9)	3 (3)	11 (9)	5 (3)	8 (7)	4 (17)	2 (6)	
Other glomerulonephritis	39 (6.5)		10 (15)	8 (9)	2 (2)	7 (4)	5 (5)	1 (4)	6 (17)	
Renovascular nephropathy	10 (2)		2 (3)	1 (1)	0 (0)	4 (2)	3 (3)	0 (0)	0 (0)	
Non-recovery from AKI	18 (3)		1 (2)	5 (6)	4 (3)	7 (4)	1 (1)	1 (4)	0 (0)	
Toxic	33 (5.5)		0 (0)	2 (2)	10 (8)	11 (7)	9 (8)	1 (4)	0 (0)	
Other or unknown	79 (13)		8 (12)	10 (11)	16 (13)	17 (11)	11 (10)	3 (13)	14 (40)	
**Medications**, n (%)										
ACEIs and/or ARBs	401 (64)	2 (6.5)^¥^	43 (66)	57 (65)	84 (70)	111 (70)	78 (72)	13 (56)	13 (37)	**p** **<** **0.05**
Diuretics	243 (39)	3 (9.7)^¥^	12 (18)	30 (34)	37 (31)	74 (47)	66 (61)	12 (52)	9 (26)	**p** **<** **0.05**
Calcium channel blockers	226 (36)	1 (3.2)^¥^	10 (15)	24 (27)	44 (37)	74 (47)	56 (52)	11 (48)	6 (17)	**p** **<** **0.05**
Beta-blockers	280 (45)	2 (6.5)^¥^	9 (14)	22 (25)	60 (50)	100 (63)	70 (65)	6 (26)	11 (31)	**p** **<** **0.05**
Other antihypertensive drugs	83 (13)	0 (0.0)^¥^	2 (3)	60 (7)	12 (10)	28 (18)	25 (23)	6 (26)	4 (11)	**p** **<** **0.05**
Statins	340 (54)	1 (3.2)^¥^	18 (28)	39 (44)	68 (57)	112 (71)	76 (70)	16 (70)	10 (29)	**p** **<** **0.05**

BMI: body mass index; ADPKD: autosomal dominant polycystic kidney disease; ACEI: angiotensin-converting-enzyme inhibitor; ARB: angiotensin II receptor blocker.

Continuous variables are expressed as the median [IQR] and binary variables are expressed as the number (%).

*The p value is for the comparison between CKD groups. Healthy controls vs CKD patients: ^¥^p < 0.001.

The median age was 64 years, 58.1% of the participants were male, 29% had diabetes mellitus, and 85.5% had hypertension. The groups differed with regard to age; controls, CKD G1 and G2 groups being significantly younger than CKD G4 and G5 groups. There were no statistically significant intergroup differences in gender or ethnicity. The mean arterial pressure was higher in the CKD G2 to G5 groups than in the other groups. Prevalence of diabetes was higher in the CKD G3B, CKD G4 and CKD G5 groups.

We observed statistically significant intergroup differences for serum phosphate, C-reactive protein (CRP), hemoglobin, glucose and lipid parameters and percentage of patients with significant proteinuria (>200 mg/L) (Supplementary Table 2).

### Relationship between eGFR and miRNA baseline levels

**Figure 2:**
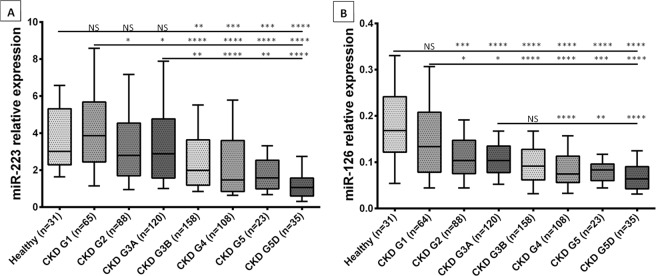
Relative expression (serum levels) of miRNAs in healthy controls, patients at different CKD stages, and patients on RRT. (**A**) Serum levels of miR-223. (**B**) Serum levels of miR-126. Data are shown as the median with box and whisker plots, including the 10^th^ and 90^th^ percentiles. *p < 0.05, **p < 0.01, ***p < 0.001, ****p < 0.0001 (in a Mann-Whitney test).

The two miRNAs were detected in all samples. The serum levels of miR-126 and miR-223 over the course of CKD are summarized in Fig. 2.

The serum levels of miR-223 were significantly lower in the CKD G3B, CKD G4, CKD G5 and CKD G5D groups than in the healthy controls and the CKD G1 and CKD G3A groups. There were no statistically significant differences between the control group on one hand, and the CKD G1, CKD G2, and CKD G3A groups on the other.

When compared with controls, serum levels of miR-126 were significantly lower in the CKD G2, CKD G3A, CKD G3B, CKD G4, CKD G5 and CKD G5D groups but not in the CKD G1 group.

The correlation between serum miR-126 and miR-223 levels in the cohort is presented in Supplementary Figure 1 and is statistically significant. Serum levels of miR-126 and miR-223 were the same whatever dialysis technique (Supplementary Figure 2). It should be noted that the exogenous control used here (Cel-miR-39) had a similar Cq for all groups (Supplementary Figure 3).

### Association of miR-126 and miR-223 with clinical and laboratory parameters

The results of linear regression analysis between miR-126 or miR-223 and baseline clinical and laboratory parameters are presented in Table 2.

**Table 2:** Association of miR-223 and miR-126 with clinical and laboratory parameters (Spearman’s rank correlation coefficient, ρ).

Parameters	miR-223		miR-126	
	ρ	p	ρ	p
Age (n = 628)	−0.21	**p < 0.0001**	−0.28	**p < 0.0001**
Sex (ref:female) (n = 628)	0.15	**p = 0.0001**	0.01	p = 0.78
Hypertension (n = 627)	−0.05	p = 0.19	−0.18	**p < 0.0001**
MAP (n = 627)	−0.02	p = 0.60	−0.02	p = 0.66
BMI (n = 625)	−0.10	**p = 0.009**	−0.13	**p = 0.0008**
Diabetes (n = 628)	−0.08	**p = 0.04**	−0.09	**p = 0.03**
CRP (n = 591)	−0.03	p = 0.43	−0.04	p = 0.28
Serum Creatinine (n = 562)	−0.31	**p < 0.0001**	−0.23	**p < 0.0001**
eGFR (n = 562)	0.30	**p < 0.0001**	0.26	**p < 0.0001**
Urea (n = 549)	−0.29	**p < 0.0001**	−0.25	**p < 0.0001**
Calcium (n = 552)	0.09	**p = 0.03**	−0.02	p = 0.68
Phosphate (n = 548)	−0.09	**p = 0.04**	−0.17	**p < 0.0001**
PTH (n = 438)	−0.26	**p < 0.0001**	−0.23	**p < 0.0001**
25-OH-D (n = 330)	−0.08	p = 0.17	−0.09	p = 0.12
Proteinuria (n = 524)	−0.07	p = 0.19	−0.10	**p = 0.02**
Hemoglobin (n = 583)	0.29	**p < 0.0001**	0.24	**p < 0.0001**
Leukocyte count (n = 581)	0.31	**p < 0.0001**	0.04	p = 0.28
Platelet count (n = 580)	0.24	**p < 0.0001**	0.18	**p < 0.0001**
Glucose (n = 537)	−0.04	p = 0.31	−0.08	p = 0.08
Triglycerides (n = 518)	−0.03	p = 0.44	0.01	p = 0.91
Cholesterol (n = 527)	0.16	**p = 0.0002**	0.14	**p = 0.0009**
HDL (n = 440)	0.08	p = 0.11	0.06	p = 0.22
LDL (n = 422)	0.15	p = 0.002	0.13	p = 0.006

MAP: mean arterial pressure; BMI: body mass index; CRP: C-reactive protein; eGFR: estimated glomerular filtration rate; PTH: parathyroid hormone; 25-OH-D: 25-hydroxy vitamin D; HDL: high-density lipoprotein; LDL: low-density lipoprotein.

The healthy control group was excluded from the analysis of laboratory characteristics and the RRT group was excluded for non-interpretable laboratory parameters (serum calcium, phosphate, urea, eGFR and proteinuria), due to the dialysis.

A multiple regression analysis was used to assess the relationship between miR-223 and the parameters significantly associated with miRNA expression in a univariate analysis. Parameters independently associated with miR-223 levels were the leukocyte count, eGFR, hemoglobin and sex (Supplementary Table 3).

Similarly, we used a multiple linear regression model of the entire cohort that included the variables significantly associated with miR-126 expression in univariate analysis; only eGFR, platelet count, hemoglobin level, and age were independently associated with serum miR-126 levels (Supplementary Table 3).

### All-cause mortality and cardiovascular outcomes

**Figure 3:**
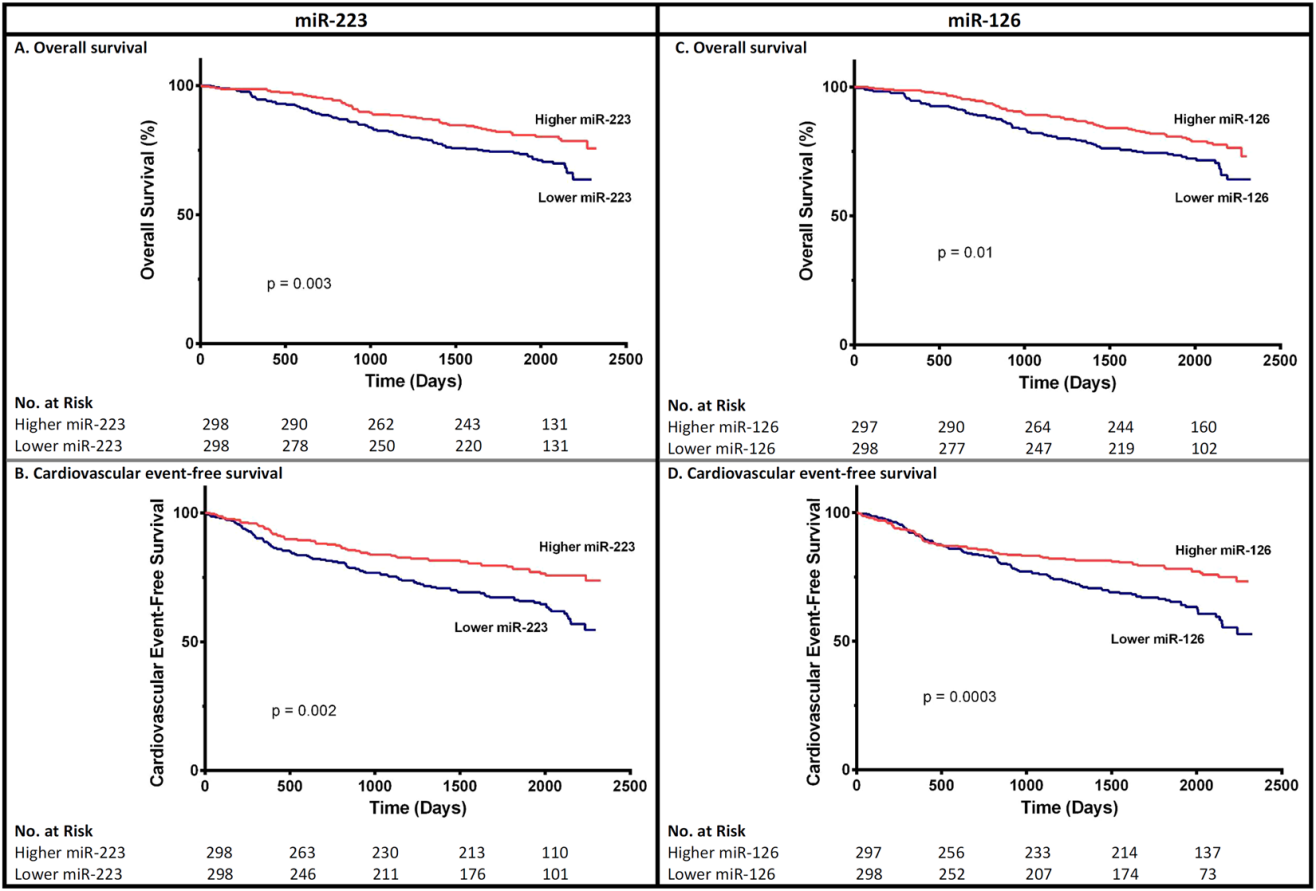
Unadjusted survival curves for primary outcomes for participants with above-median and below-median serum miR-223 (**A** and **B**) or miR-126 (**C** and **D**) levels (Log-rank test).

Overall, during a median follow-up period of 1948 days [1630–2146], 173 patients experienced cardiovascular events and 150 died (Table 3). Infection accounted for 25 deaths, malignant disease for 24, and cardiovascular events for 29 deaths (Table 3). Twelve patients were lost to follow-up. When considering all-cause mortality, patients with levels of miR-223 and miR-126 below the median had a lower survival rate (Fig. 3A and C). However, the associations between both miRNA levels and overall mortality were no longer significant after adjustment for baseline eGFR (Supplementary Table 4). To confirm this result, we performed a power analysis. We assume a one-sided adjusted alpha risk of 2.5% (two miRNA were studied), a power of 80% and a correlation coefficient of 0.35 between eGFR and each miRNA. With the latter hypotheses, 150 deaths could permit the detection of a HR = 1.63 between the two groups defined by the median miRNA. An HR around 1.50 is realistic for a superiority time-to-event trial, therefore our study had enough statistical power to compared overall survival according to miRNA. Sensitivity analysis using Cox models adjusted for each CKD stage or a model with the eGFR*miR interaction or combining both miRNAs levels did not reveal any significant associations between miRNA levels and mortality after adjustment for eGFR baseline either (data not shown). When we focused more specifically on cardiovascular mortality, there was no significant association with miRNA levels after adjustment for baseline eGFR (Supplementary Table 5). When considering CV events, patients with below-median levels of miR-223 and miR-126 had a lower CV event rate (Fig. 3B and D). However, theses associations were no longer significant after adjustment for baseline eGFR (Supplementary Table 6).

**Table 3:** Outcomes in the CKD cohort and each CKD stage group.

n	CKD Cohort	CKD G1	CKD G2	CKD G3A	CKD G3B	CKD G4	CKD G5	CKD G5D	p
	597	65	88	120	158	108	23	35	
Follow-up time (days)	1948 [1630–2146]	1988 [1744–2181]	1966 [1787–2142]	2034 [1735–2182]	1920 [1417–2117]	1869 [938–2130]	1907 [775–2171]	1839 [1135–2157]	p = 0.006
Alive, n (%)	447 (75)	64 (98)	79 (90)	99 (82)	112 (71)	58 (54)	13 (56)	22 (63)	p < 0.0001
Primary outcome, n (%)	250 (42)	4 (6)	16 (18)	43 (36)	85 (54)	65 (60)	14 (61)	23 (66)	p < 0.0001
Death, n (%)	150 (25)	1 (2)	9 (10)	21 (17)	46 (29)	50 (46)	10 (43)	13 (37)	p < 0.0001
infection	25 (4)	1 (2)	0 (0)	3 (3)	6 (4)	11 (10)	1 (4)	3 (9)	
malignancy	24 (4)	0 (0)	0 (0)	6 (5)	9 (6)	7 (6)	1 (4)	1 (3)	
cardiovascular cause	29 (5)	0 (0)	3 (3)	3 (3)	11 (7)	9 (8)	1 (4)	2 (6)	
refuse dialysis	4 (1)	0 (0)	0 (0)	0 (0)	0 (0)	2 (2)	0 (0)	2 (6)	
undetermined	68 (11)	0 (0)	6 (7)	9 (8)	20 (13)	21 (19)	7 (30)	5 (14)	
Cardiovascular events, n (%)	173 (29)	3 (5)	11 (12)	33 (27)	63 (40)	39 (36)	8 (35)	16 (46)	p < 0.0001
atheromatous	119 (20)	3 (5)	6 (7)	27 (22)	41 (26)	26 (24)	6 (26)	10 (29)	
non-atheromatous	71 (12)	0 (0)	6 (7)	9 (7)	26 (16)	20 (19)	3 (13)	7 (20)	
Secondary outcome, n (%)*	83 (15)	0 (0)	7 (8)	6 (5)	18 (11)	38 (35)	14 (61)	n.a.	p < 0.0001
Renal events, n (%)	57 (10)	0 (0)	3 (3)	1 (1)	7 (4)	32 (30)	14 (61)	n.a.	p < 0.0001
dialysis	49 (9)		3 (3)	1 (1)	7 7 (4)	27 (25)	11 (48)		p < 0.0001
transplant	16 (3)		0 (0)	0 (0)	0 (0)	6 (6)	5 (22)		p < 0.0001
Renal impairment, n (%)	45 (8)	0 (0)	6 (7)	6 (5)	13 (8)	18 (17)	2 (9)	n.a.	p < 0.0001

n.a.: not applicable.

*n = 562 (excluding CKD 5D).

Continuous variables are expressed as the median [IQR] and binary variables are expressed as the number (%).

We further divided CV events into atheromatous and non-atheromatous events and performed the statistical analysis for each in relation with the miRNA levels. The results were globally similar, but it should be pointed out that in univariate analysis, miR-223 was associated with non-atheromatous events, but not with atheromatous events (Supplementary Tables 7 and 8).

### Renal outcomes

**Figure 4:**
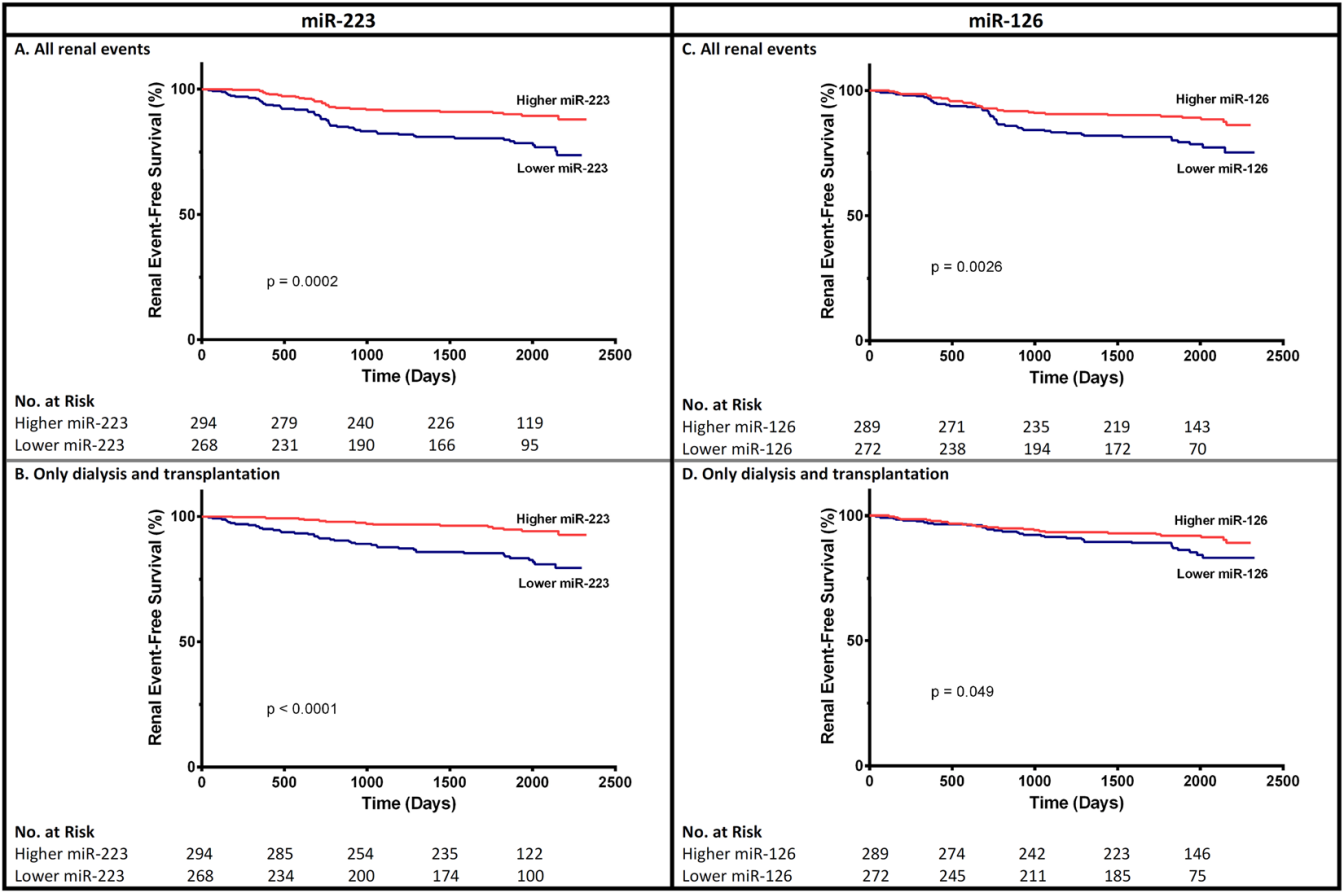
Unadjusted survival curves for secondary outcomes for participants with above-median and below-median serum miR-223 (**A** and **B**) or miR-126 (**C** and **D**) levels (Log-rank test).

Among the 562 non-dialysis CKD patients, 57 developed need for renal replacement therapy, either dialysis or transplantation during follow-up (Table 3) and 45 experienced an accelerated loss of renal function (defined as a doubling of the serum creatinine level, or a reduction of eGFR by 30%, over two years). CKD patients stage G1 to G5 not yet on dialysis with above-median levels of miR-223 and miR-126 had a significantly better event-free survival rate (Fig. 4). However, the associations between both miRNA levels and renal events were no longer significant after adjustment for baseline eGFR (Supplementary Table 9).

After a median follow up of 737 days, among 458 patients with non-dialysis CKD, 134 patients were considered as fast progressors (an eGFR decrease of more than 3 mL/min/1.73 m²/year), 229 as non-progressors (an eGFR decrease of less than 1 mL/min/1.73 m²/year) and 95 as slow progressors (an eGFR decrease of between 1 and 3 mL/min/1.73 m²/year). There was no difference between the three groups with regard to the serum levels of miR-223 and miR-126 (Supplementary Figure 4).

## Discussion

The present study demonstrated a statistically significant decrease in circulating miR-126 and miR-223 levels in patients with more severe stages of CKD. Levels of miR-126 were independently associated with eGFR but also with hemoglobin level, platelet count, and age. The circulating level of miR-223 was independently associated with leukocyte count and hemoglobin level, sex, and eGFR. We did not find an independent correlation between any studied miRNA and the serum level of CRP or with diabetes. The event-free survival rate was better in patients with higher circulating miRNA levels. After adjustment for eGFR, neither miR-126 nor miR-223 level were prognostic markers of all-cause mortality, cardiovascular events or renal events. We did not observe an association between the initial circulating levels of miR-126 and miR-223 and the decline of kidney function over two years.

Glomerular filtration rate estimated from the serum level of creatinine currently remains the most convenient marker of renal function for everyday practice and large epidemiologic studies. Albuminuria, proteinuria and seric urea levels are useful indicators of renal function when normalized for urinary creatinine. They are however not very sensitive when used to detect the first stages of CKD^33^. Several other markers have been assessed for their predictive value of mortality, cardiovascular (CV) complications and kidney disease progression without success^34–40^. miRNAs have recently attracted attention as biomarker candidates to assess the severity and/or the etiologies of kidney disease^41,42^. One of the main advantages of miRNAs is their seric stability, which makes them suitable as a non-invasive biomarker^27^. One can thus hope that these small RNAs could prove to be reliable enough to be useful in potential clinical practice^43,44^.

Quantitative PCR is a technique commonly used for accurate quantification of miRNA levels in blood samples and transplantable in a clinical laboratory. In quantitative PCR, it is important to choose an adequate control gene, especially as miRNA levels are low in the circulation. Some teams have chosen to use small endogenous RNAs, such as U6, to normalize circulating miRNAs levels but the results have proven to be unreliable due to marked variability of the expression of these RNAs according to pathological conditions^45^. An emerging consensus is now to spike-in in the blood sample before extraction a given quantity of exogenous miRNA (such as the synthetic *Caenorhabditis elegans* miR-39)^46,47^.

Several teams have shown that the overall expression of miRNAs blood concentration diminishes as CKD advances^48,49^. In a cohort of 90 CKD patients (stages G3 to G5D) Chen *et al*. published that miR-125b, miR-145 and miR-155 levels decline as CKD progresses^50^. The blood levels of cardiac miRNAs also seems to decline with eGFR^51^. In the present study, we observed a decrease in serum levels of miR-126 and miR-223 as renal function declined. However, proof that kidney function affects miRNA levels in plasma and urine is sparse and sometimes contradictory. Some studies were interested in the possible accumulation of RNases known in renal failure^52^ which could increase degradation of circulating miRNAs in the plasma, but these are protected in particular by exosomes and Argonaut proteins. In the present study, serum RNA levels after extraction did not differ as a function of the CKD stage (data not shown) – suggesting that there is no degradation, in line with other reports^50^.

Over the last years, we studied the role of uremic toxins in *in vitro* models of osteoclastogenesis and vascular calcification.^5^ We highlighted the roles of miR-223 and miR-126 in the trans-differentiation of VSMCs to an osteoblastic phenotype linked with vascular calcification^18,53^. We confirmed this finding *in vivo* by showing that miR-126 and miR-223 expression was enhanced in aortas of murine CKD models, while their serum levels were decreased^8^ which may indicate an accumulation of these two miRNAs in vascular walls. In 2016, Ulbing *et al*. found a decrease of seric levels of miR-223 in CKD patients that was alleviated after renal graft^19^. In our large cohort covering the whole spectrum of CKD, we also observed low expression - from stage G3B onwards for miR-223, and from stage G2 onwards for miR-126.

In the present study, the eGFR was the clinical feature most strongly correlated with miRNA concentration (apart from leukocyte count for miR-223 and age for miR-126). The observed associations between the miRNA level and the other features of severe kidney failure (e.g. hyperparathyroidism and hyperphosphatemia) in univariate analyses were mediated by the impairment in glomerular filtration. There was a good degree of correlation between miR-223 level on one hand and sex, leukocyte count, platelet count, and blood hemoglobin and cholesterol levels on the other. The relationship with blood cell counts has already been reported, and is related to the involvement of miR-223 in hematopoiesis and its strong expression in granulocytes, platelets and red blood cells^10–12,54–56^. miR-223 has been involved before in cholesterol homeostasis^57^, and we found accordingly a correlation with cholesterol levels using univariate analysis. In our multivariate analysis, only loss of kidney function, a low leukocyte count, a low hemoglobin level, and male sex were associated with low circulating levels of miR-223. Although miR-223 is often considered to be a marker of inflammation^58^, levels were not correlated with serum CRP. In univariate analyses, the circulating level of miR-126 was notably correlated with age, hemoglobin and cholesterol levels, and platelet count. The correlation with cholesterol and LDL has already been reported but the underlying mechanism have not yet been identified^59^. In our multivariate analysis, miR-126 level was independently associated with eGFR, platelet count, hemoglobin level, and age. The correlations with hemoglobin and eGFR have previously been described^60^, as have the correlations with platelet count^61^ and age^62^. Grabher *et al*. demonstrated that miR-126 regulates the proto-oncogene c-Myb during hematopoiesis^63^.

According to our results, miR-126 and miR-223 cannot be used as prognostic markers because of their eGFR-related variability. Indeed, miR-126 and miR-223 levels were not independently associated with poor survival after adjustment for eGFR. The results were similar for mortality, cardiovascular events, and renal events – none of which were predictive of a further decline in eGFR.

The present study is the first having investigated these two miRNAs as potential biomarkers in CKD. Its strengths include the large study population and the availability of data over the full range of CKD stages including a group of controls without CKD, enabling stratification by CKD stage. We also assessed a number of important, potentially confounding routine laboratory parameters with a long term follow-up. We studied for the first time in a large CKD cohort the impact of serum miRNA levels on hard outcomes. However, our study also has several limitations. Estimated GFR was used in the analysis, instead of the more accurate direct measurement of GFR. Nevertheless, we used the CKD-EPI formula which estimates GFR more accurately compared to other widely-used formulas. Another limitation is that miRNA levels were only measured at the baseline examination.

In conclusion, lower circulating levels of miR-126 and miR-223 were associated with lower eGFR in a cohort of patients with CKD at different stages. The observed link between the two miRNAs’ seric levels and mortality, cardiovascular events or renal events in CKD appears to depend on eGFR. However, this does not preclude their potential role in the pathophysiology of CKD, which will need further evaluation.

## Methods

### The study population

A total of 601 patients with CKD stages G1 to G5 (as defined by the Kidney Disease Outcomes Quality Initiative guidelines (KDOQI)) or on renal replacement therapy (RRT: hemodialysis or peritoneal dialysis) and 31 healthy controls were included in this single-center study at the outpatient clinic of the Section of Nephrology, Ghent University Hospital (Ghent, Belgium). Inclusion took place between January 2011 and January 2014. The exclusion criteria were pregnancy, age under 18, active infection, active malignancy or history of transplantation. Outcome parameters were monitored until June 2017 (for up to 6 years). Patients with CKD were categorized into subgroups according to their estimated glomerular filtration rate (eGFR), using the Chronic Kidney Disease Epidemiology Collaboration (CKD-EPI) – creatinine equation, as recommended by the KDOQI. Eight subgroups were compared, including six CKD subgroups not on dialysis (eGFR: over 90, 60–89, 45–59, 30–44, 15–29, and below 15 ml/min/1.73 m²), one CKD subgroup on RRT (stage G5D), and healthy controls. Hypertension was defined as a systolic blood pressure >140 mmHg and/or a diastolic blood pressure >90 mmHg, or the need for anti-hypertensive medication.

### Ethical approval

This study was approved by the local ethical committee at Ghent University Hospital. Written informed consent was obtained from all participants. The study complied with the tenets of the Declaration of Helsinki and its amendments.

### Blood samples

A sample of venous blood was collected in Venosafe serum (Terumo Europe, Leuven, Belgium) tubes. The blood was left to coagulate for 30 minutes on the bench and was then centrifuged at 2,095 × g for 10 min at room temperature (RT). The serum samples were divided into 500 μL aliquots on ice and stored at −80 °C until batch analysis.

### RNA extraction

Total RNA was extracted with the miRNeasy Serum/Plasma kit (Qiagen, Germany), according to the manufacturer’s instructions. Stored serum was thawed on ice and shaken. Next, 1000 μl of Qiazol lysis reagent was added to 200 μl of serum and incubated at RT for 5 min. A fixed amount of synthetic *Caenorhabditis elegans* miR-39 (3.5 µL of Cel-miR-39 at 1.6 × 10^8^ copies/µL, i.e. 1 fmol) was added as an internal control. Next, 200 μl of chloroform was added, and the sample was shaken for 15 s. After incubation for 3 min at room temperature, the samples were centrifuged for 15 min at 16,100 × g at 4 °C. The upper aqueous phase (approximately 700 μl) was transferred to a new tube, and 1000 μl of 100% ethanol was added. The RNA was isolated with a miRNeasy MinElute spin column and solutions (Qiagen, Germany), eluted from the columns with 14 μl RNase-free water, and stored at −80 °C.

The purity and concentration of the isolated RNA were determined using a NanoDrop spectrophotometer (Thermo Fisher Scientific, USA).

### Reverse transcription

Isolated RNA was reverse-transcribed into complementary DNA using TaqMan miRNA-specific primers and the TaqMan microRNA reverse transcription kit (Applied Biosystems, USA) on ice. Each well contained the following mixture: 2.8 μL of RNase-free water, 1.5 μL of 10X RT buffer, 1.5 μL of dNTP mix (10 mM), 0.2 μL of RNase inhibitor (20 U/μL), 1 μL of MultiScribe® RT enzyme ΜL (50 U/µL), 3 μL of 5X primers (i.e. for miR-223, miR-126 or Cel-miR-39) and 5 μL of RNA 5 ng/μL (i.e. 25 ng). The Eppendorf Mastercycler® thermocycler was used to incubate the samples at 16 °C for 30 min, at 42 °C for 30 min and then at 85 °C for 5 min. The cDNA samples were then stored at −20 °C.

### Quantification of serum miRNA levels

Serum levels of miR-223 and miR-126 were quantified using TaqMan qRT-PCR. cDNA (2.4 μL) was amplified using 18 μL of SsoAdvanced™ Universal Probes Supermix (Bio-Rad Laboratories, USA), 1.8 μL of 20X Primers and 13.8 μL of RNase-free water, in a final volume of 36 μL. All reactions and analyses were performed in triplicate on CFX Connect (Bio-Rad) according to the following protocol: incubation at 95 °C for 30 seconds, then 40 cycles of 15 seconds at 95 °C, and incubation at 60 °C for 30 seconds. Exogenously added *Caenorhabditis elegans*-miR-39 (Cel-miR-39) was used as a spiked-in normalization control. The relative expression levels of miR-223 and miR-126 were calculated using the 2-ΔCq method, where ΔCq = Cq (miR-223 or miR-126) − Cq (cel-miR-39). The maximum accepted coefficient of variation for intra-assay replicates was set to 5%. All experiments were performed by the same person (O.F.) who was blinded for the outcomes, to avoid bias.

### Outcomes

All the outcomes were collected prospectively. The primary outcomes were all-cause mortality, and cardiovascular events (fatal and non-fatal). Cardiovascular events were defined as atheromatous or non-atheromatous cardiovascular disease (CVD). Atheromatous CVD was defined as coronary artery disease (CAD), ischemic stroke or transient ischemic attack, peripheral arterial disease requiring a revascularization procedure or an amputation. Non atheromatous CVD was defined as heart failure in the absence of CAD, cardiac rhythm or conduction disorders, or aortic aneurysm.

Renal events were defined as progression towards RRT (dialysis or renal transplantation), or a doubling of the serum creatinine level, or a reduction of eGFR by 30%, over two years (according to the FDA criteria^64^). The CKD G5D group was excluded from the analysis of the renal events. The variation of eGFR between inclusion and end of renal follow-up (at one or two years of follow-up according to the patients) was calculated. We also studied miRNA levels in three strata: a decrease in the eGFR <1 mL/min/1.73 m²/year (non-progressors), 1–3 mL/min/1.73 m²/year (slow progressors) or >3 mL/min/1.73 m²/year (fast progressors).

### Statistical analyses

Patient characteristics and the relative expression levels of miR-223 and miR-126 are presented as median [interquartile range (IQR)] for continuous variables (because the data were not normally distributed except for hemoglobin) or as the number (frequency) for binary variables. Intergroup comparisons were performed using a Kruskal-Wallis test for continuous variables and a χ² test for binary variables. Comparisons between two groups were performed with a Mann-Whitney test. The relationships between patient characteristics and miRNA levels were explored using Spearman’s rank correlation tests. Multiple linear regression analyses were performed to investigate the association between clinical/laboratory parameters and the expression of miRNAs. The Kaplan-Meier survival curves were constructed to estimate overall mortality, cardiovascular and renal events by dividing the studied population according to the selected cut-off (the median level: 2.28 for miR-223 and 0.09 for miR-126). The log-rank test was used to compare survival curves. Univariate and multivariate analyses of outcomes were performed by building a Cox proportional hazards model based on miRNA levels. Due to non-normal distribution, the data were log-transformed. The multivariate analysis included all the parameters with a p-value < 0.05 in the univariate analysis. In all the tests, the threshold for statistical significance was set to p < 0.05. Statistical analyses were performed using GraphPad Prism® software version 6 and SPSS® software version 21. The power analysis was performed using package powerSurvEpi with software “RStudio software Version 1.0.143 – © 2009–2016 RStudio (R.3.4.0 software)” as described previously^65^.

## Supplementary information

Supplementary Figures and tables
