# Supplementary material for: Serum levels of miR-126 and miR-223 and outcomes in chronic kidney disease patients
Source: Sci Rep. 2019 Mar 14;9:4477. doi: 10.1038/s41598-019-41101-8 (PMC6418179; doi:10.1038/s41598-019-41101-8)
Supplement: Supplementary file 1 — Supplementary Figures and tables [file 41598_2019_41101_MOESM1_ESM.pdf]

**Serum levels of miR-126 and miR-223 and outcomes in chronic kidney disease patients**

Ophélie Fourdinier[1,2,*], MSc, Eva Schepers[3,*], PhD, Valérie Metzinger-Le Meuth[1,4], PhD, Griet Glorieux[3], PhD, Sophie Liabeuf[1,5], PharmD-PhD, Francis Verbeke[3], MD-PhD, Raymond Vanholder[3], MD-PhD, Benjamin Brigant[1,6], MSc, Anneleen Pletinck[3], PhD, Momar Diouf[7], PhD, Stéphane Burtey[8], MD-PhD, Gabriel Choukroun[1,2], MD-PhD, Ziad A. Massy[9], MD-PhD, Laurent Metzinger[1,6], PharmD-PhD, on behalf of the European Uremic Toxin Work Group-EUTox

Supplementary files

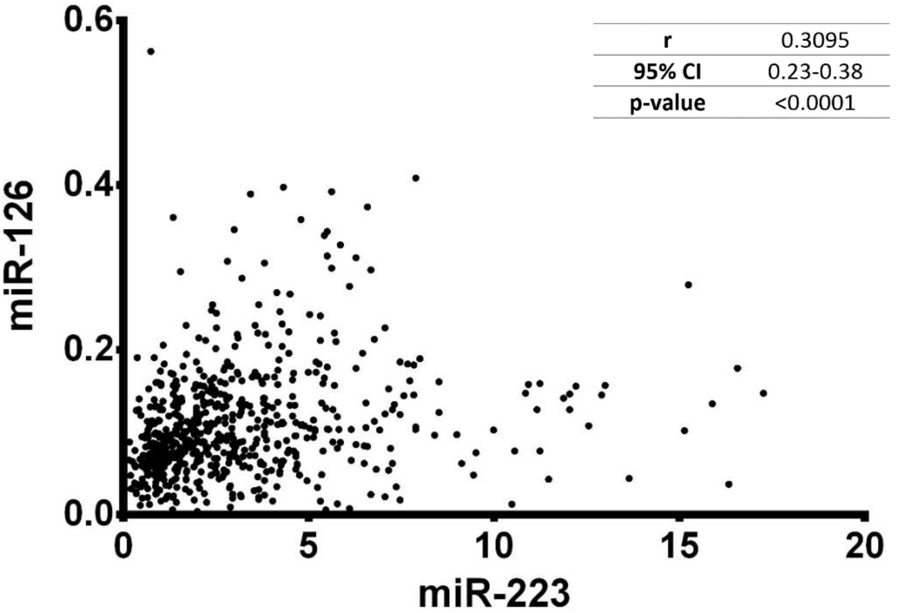


**Supplementary Figure 1. Correlation between serum miR-126 and miR-223 levels in the cohort as a whole. Spearman's correlation test.**

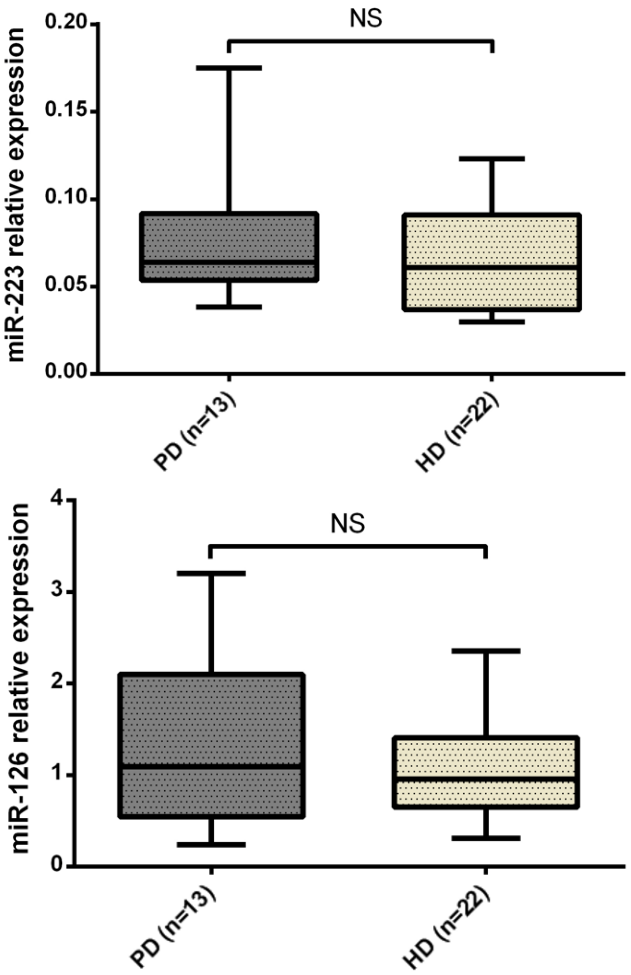


**Supplementary Figure 2. miRNA relative expression according to dialysis technique**
PD: peritoneal dialysis; HD: hemodialysis.

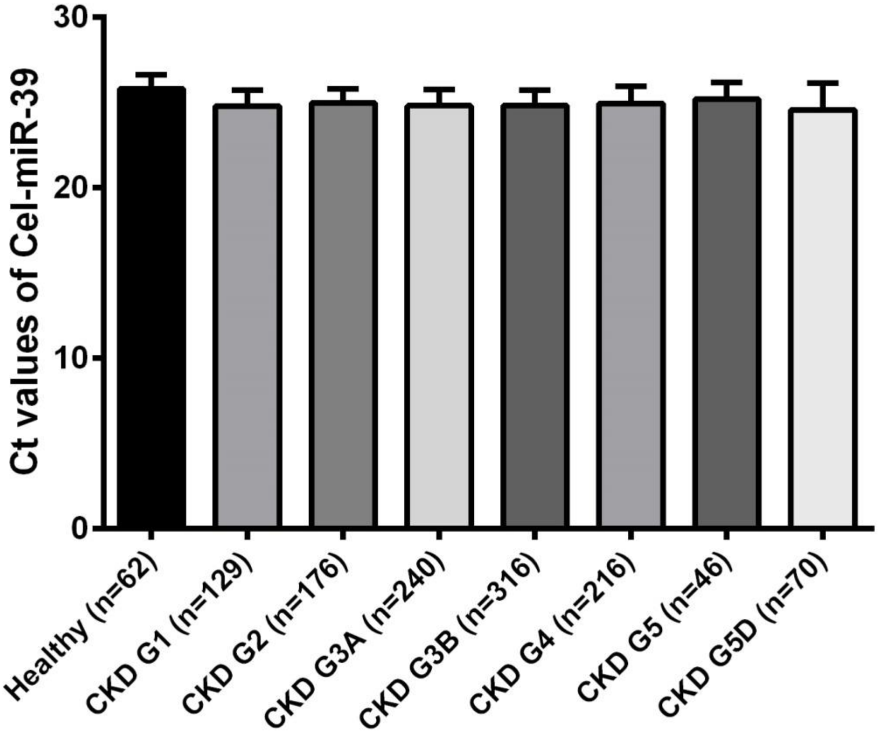


**Supplementary Figure 3. Mean Ct values for Cel-miR-39 as a function of the CKD stage.** Data are quoted as the mean ± SEM.

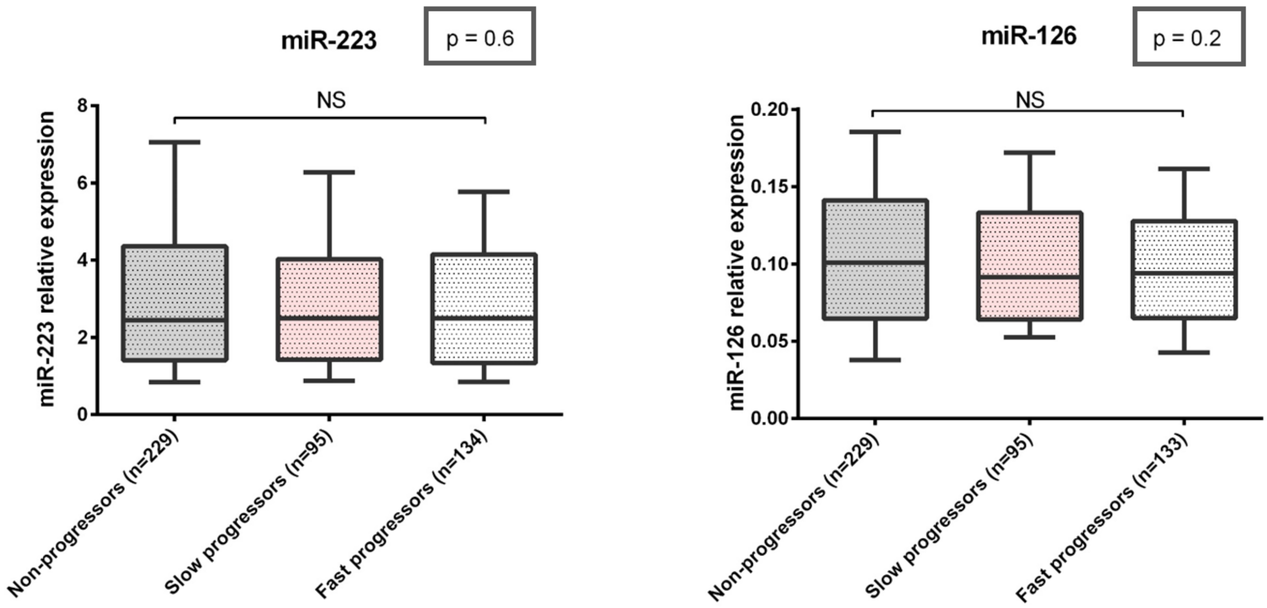


**Supplementary Figure 4. Relative expression (serum levels) of miR-223 and miR-126 in CKD patients, as a function of eGFR progression**

NS: non-significant.

*Non-progressors: eGFR decrease < 1 mL/min/year; slow progressors: eGFR decrease between 1 and 3 mL/min/year; fast progressors: eGFR decrease > 3 mL/min/year* (after exclusion of the RRT group).

Data are shown as the median with box and whisker plots, including the $10^{th}$ and $90^{th}$ percentiles.

Comparisons were performed using a Mann-Whitney test.

**Supplementary Table 1. Validated human targets of miR-223 and miR-126, according to miRTarBase**

| miR-223 | | miR-126 | |
|---|---|---|---|
| LMO2 | SEPT2 | TOM1 | SLC41A2 |
| NFIA | POLR3G | CRK | GRIN2B |
| MEF2C | FOXO3 | VEGFA | EVI5 |
| STMN1 | CDC27 | IRS1 | PIK3R1 |
| RHOB | SP1 | SOX2 | SZRD1 |
| IGF1R | CCL3 | TWF1 | L2HGDH |
| FBXW7 | IL6 | PITPNC1 | PLAGL2 |
| CHUK | CXCL2 | IGFBP2 | |
| PARP1 | BAG2 | KRAS | |
| PRDM1 | NSUN3 | SPRED1 | |
| CARM1 | MTRF1L | PLK2 | |
| MAFB | RIF1 | EGFL7 | |
| E2F1 | TP53 | RGS3 | |
| Arid4b | TMEM64 | HOXA9 | |
| Lpin2 | SLC7A5 | PIK3R2 | |
| ARTN | SESN3 | TWF2 | |
| FOXO1 | RRAS2 | DNMT1 | |
| CDK2 | NOVA2 | SLC7A5 | |
| TAL1 | LATS2 | PIK3CG | |
| ATM | F3 | ADAM9 | |
| CYB5A | MDM2 | CRKL | |
| TOX | MKNK2 | BCL2 | |
| MSMO1 | IL6ST | SLC45A3 | |
| ABCB1 | SPPL2A | VCAM1 | |
| CAPRIN1 | C9orf40 | PTPN7 | |
| PAX6 | ZBTB18 | TEK | |
| STAT1 | PHF19 | FOXO3 | |
| NLRP3 | CHMP2B | CXCR4 | |
| HAX1 | WASL | RHOU | |
| EPB41L3 | ARL8B | LRP6 | |
| LIF | FABP7 | SIRT1 | |
| SLC2A4 | TMEM67 | NFKBIA | |
| NFIX | PDZD8 | CADM1 | |
| SP3 | SINHCAF | EZH2 | |
| HSP90B1 | ITGB1 | ROCK1 | |
| SMARCD1 | NMNAT2 | CCNE2 | |
| ECT2 | RANGAP1 | E2F1 | |
| PTBP2 | GPATCH8 | PGR | |
| NAMPT | CACNG8 | ADGRE5 | |
| SNX24 | ZNF460 | AKT1 | |
| CDS1 | PAX4 | MMP7 | |
| SECISBP2L | HEXIM1 | CXCL12 | |
| TWF1 | TRPV2 | RBMX | |
| ZNF365 | MYL9 | Camsap1 | |
| STAT5A | ZEB1 | TCF4 | |
| CFTR | POTEG | Cdkn1b | |
| STAT3 | POTEM | ADM | |
| SEMA3A | PRRC2C | KCNJ1 | |
| IRS1 | SLC12A7 | SLC39A6 | |
| SCARB1 | SYNGR2 | AKT2 | |
| Hnf1a | LGALS8 | ACVR2B | |

**Supplementary Table 2: Baseline laboratory parameters as a function of the CKD stage**

| | CKD G1 | CKD G2 | CKD G3A | CKD G3B | CKD G4 | CKD G5 | CKD G5D | P value* |
|---|---|---|---|---|---|---|---|---|
| n | 65 | 88 | 120 | 158 | 108 | 23 | 35 | |
| Serum Creatinin, µmol/L | 68.9 [62.8-76.9] | 91.9 [78.7-103.4] | 115.4 [101.7-127.1] | 147.6 [133.3-163.8] | 215.3 [192.0-254.6] | 351.8 [305.0-448.2] | n.a. | n.a. |
| eGFR (CKD-Epi), ml/min/1.73m² | 105.6 [96.6-115.0] | 71.4 [65.4-81.8] | 50.4 [47.8-55.2] | 36.5 [34.2-39.8] | 24.5 [19.1-27.6] | 12.9 [11.1-13.7] | n.a. | n.a. |
| eGFR (MDRD), ml/min/1.73m² | 98.3 [87.4-106.4] | 67.0 [61.6-73.7] | 49.9 [47.0-52.9] | 37.1 [34.3-40.1] | 25.2 [20.4-28.1] | 13.5 [12.0-14.1] | n.a. | n.a. |
| Urea, mmol/L | 5.0 [4.3-5.8] | 6.6 [5.3-7.8] | 8.0 [6.8-9.3] | 11.0 [9.1-13.6] | 15.8 [13.3-19.4] | 22.6 [20.3-25.1] | n.a. | n.a. |
| Calcium, mmol/L | 2.35 [2.3-2.4] | 2.38 [2.3-2.4] | 2.38 [2.3-2.4] | 2.35 [2.3-2.4] | 2.35 [2.3-2.4] | 2.3 [2.2-2.5] | n.a. | p = 0.55 |
| Phosphate, mmol/L | 1.02 [1.0-1.2] | 0.99 [0.9-1.1] | 1.06 [0.9-1.2] | 1.09 [1.0-1.2] | 1.12 [1.0-1.3] | 1.44 [1.2-1.6] | n.a. | **p < 0.05** |
| Proteinuria, % | 25.6 | 26.2 | 18.9 | 32.4 | 57.1 | 95.4 | n.a. | **p < 0.05** |
| CRP, mg/L | 1.0 [0.0-2.0] | 1.0 [0.0-4.0] | 2.0 [1.0-5.0] | 2.8 [1.0-5.0] | 3.0 [1.0-6.0] | 2.0 [1.0-17.0] | 4.0 [2.0-12.0] | **p < 0.05** |
| Hemoglobin, g/dL | 14.4 [13.2-15.2] | 14.2 [13.2-15.5] | 13.9 [12.6-14.8] | 13.5 [12.3-14.7] | 12.6 [11.9-13.8] | 12.1 [11.2-12.7] | 11.7 [10.9-12.6] | **p < 0.05** |
| Leukocyte count, $10^9$/L | 6.5 [5.2-7.9] | 6.4 [5.4-7.5] | 6.7 [5.2-8.3] | 6.9 [5.6-8.5] | 6.8 [5.7-8.2] | 7.2 [6.1-9.0] | 5.9 [5.1-7.6] | p = 0.11 |
| Platelet count, $10^9$/L | 241 [202-297] | 327 [208-281] | 230 [196-287] | 226 [178-277] | 219 [182-262] | 230 [182-253] | 212 [184-281] | p = 0.08 |
| Total cholesterol, mmol/L | 4.8 [4.4-5.4] | 5.1 [4.4-5.7] | 4.7 [4.1-5.3] | 4.3 [3.8-5.2] | 4.4 [3.9-5.3] | 4.7 [4.2-6.0] | 4.2 [4.0-4.9] | **p < 0.05** |
| HDL-cholesterol, mmol/L | 1.6 [1.3-2.0] | 1.6 [1.3-1.8] | 1.5 [1.3-1.9] | 1.3 [1.1-1.7] | 1.2 [1.1-1.7] | 1.4 [1.1-2.0] | 1.2 [1.0-1.4] | **p < 0.05** |
| LDL-cholesterol, mmol/L | 2.7 [2.2-3.1] | 2.6 [2.4-3.3] | 2.4 [2.0-3.0] | 2.2 [1.7-2.8] | 2.4 [1.8-3.1] | 2.7 [2.4-3.5] | 2.4 [2.0-2.9] | **p < 0.05** |
| Triglycerides, mmol/L | 1.1 [0.8-1.3] | 1.2 [0.9-1.6] | 1.3 [1.0-1.7] | 1.4 [1.0-2.0] | 1.5 [1.0-2.1] | 1.2 [1.1-1.4] | 1.6 [1.1-1.9] | **p < 0.05** |
| Glucose, mmol/L | 4.9 [4.5-5.3] | 4.9 [4.4-5.3] | 5.2 [4.7-5.8] | 5.6 [4.9-6.9] | 5.3 [4.8-6.7] | 4.8 [4.5-5.3] | n.a. | **p < 0.05** |

eGFR: estimated glomerular filtration rate; CRP: C-reactive protein; HDL: high-density lipoprotein; LDL: low-density lipoprotein; n.a. not applicable.

Continuous variables are expressed as the median [IQR] and binary variables are expressed as the number (%). * The p value is for the comparison between CKD groups.

**Supplementary Table 3. Multiple linear regression analysis of variables independently associated with circulating levels of miR-223 (A) and miR-126 (B)**

**(A)**

| **miR-223** | **β** | **95%CI** | **p-value** |
|---|---|---|---|
| **Leukocyte count** (per 1/mm$^3$) | 0.38 | [0.28-0.47] | < 0.0001 |
| **eGFR** (CKD-Epi) (per 1 mL/min/1.73m$^2$) | 0.02 | [0.01-0.03] | < 0.0001 |
| **Hemoglobin** (per 1 g/dL) | 0.35 | [0.22-0.48] | < 0.0001 |
| **Sex** (ref: female) | 0.90 | [0.45-1.35] | < 0.0001 |

β: regression coefficient; CI: confidence interval; eGFR: estimated glomerular filtration rate
$R^2$ for the model = 0.194
The initial model included miR-223 as the independent variable and all the parameters significantly associated with miR-223 in a univariate analysis.
Variables entered into the model for miR-223 and excluded: age, BMI, diabetes, urea, calcium, phosphate, PTH, platelet count, cholesterol, and LDL.

**(B)**

| **miR-126** | **β** | **95%CI** | **p-value** |
|---|---|---|---|
| **eGFR** (CKD-Epi) (per 1 mL/min/1.73m$^2$) | 0.000 | [0.000-0.001] | 0.002 |
| **Platelet count** (per 1/mm$^3$) | 0.000 | [0.000-0.000] | 0.001 |
| **Hemoglobin** (per 1 g/dL) | 0.005 | [0.002-0.008] | 0.002 |
| **Age** (per year) | 0.000 | [-0.001-0.000] | 0.026 |

β: regression coefficient; CI: confidence interval; eGFR: estimated glomerular filtration rate
$R^2$ for the model = 0.124
The initial model included miR-126 as the independent variable and all the parameters significantly associated with miR-126 in a univariate analysis.
Variables entered in the model for miR-126 and then excluded: hypertension, BMI, diabetes, urea, phosphate, PTH, proteinuria, cholesterol, and LDL.

**Supplementary Table 4. Univariate and multivariate Cox regression analyses of risk factors at baseline for all-cause mortality**

| miR-223 | | | |
|---|---|---|---|
| Models of patient survival (events = 150) | **RR** | **95% CI** | **p** |
| *Unadjusted* | | | |
| **ln miR-223** | **0.72** | **0.59-0.86** | **< 0.0001** |
| *Model adjusted for age, sex, and diabetes* | | | |
| **ln miR-223** | **0.80** | **0.66-0.98** | **0.033** |
| Age per 1 year | 1.08 | 1.06-1.10 | < 0.0001 |
| Male | 1.25 | 0.89-1.76 | 0.194 |
| Diabetes | 1.46 | 1.06-2.02 | 0.021 |
| *Model adjusted for age, sex, diabetes, and baseline eGFR* | | | |
| ln miR-223 | 0.93 | 0.75-1.16 | 0.530 |
| Age per 1 year | 1.07 | 1.05-1.09 | < 0.0001 |
| Male | 1.54 | 1.07-2.22 | 0.020 |
| Diabetes | 1.45 | 1.03-2.03 | 0.030 |
| Baseline eGFR per 1 ml/min/1.73m$^2$ | 0.98 | 0.97-0.99 | < 0.0001 |

RR: relative risk; CI: confidence interval; eGFR: estimated glomerular filtration rate.

| miR-126 | | | |
|---|---|---|---|
| Models of patient survival (events = 150) | **RR** | **95% CI** | **p** |
| *Unadjusted* | | | |
| **ln miR-126** | **0.72** | **0.57-0.91** | **0.006** |
| *Model adjusted for age, sex, and diabetes* | | | |
| ln miR-126 | 0.93 | 0.73-1.20 | 0.595 |
| Age per 1 year | 1.08 | 1.06-1.09 | < 0.0001 |
| Male | 1.29 | 0.93-1.82 | 0.133 |
| Diabetes | 1.46 | 1.06-2.02 | 0.022 |
| *Model adjusted for age, sex, diabetes, and baseline eGFR* | | | |
| ln miR-126 | 1.07 | 0.81-1.42 | 0.634 |
| Age per 1 year | 1.07 | 1.05-1.09 | < 0.0001 |
| Male | 1.56 | 1.09-2.25 | 0.016 |
| Diabetes | 1.45 | 1.03-2.02 | 0.034 |
| Baseline eGFR per 1 ml/min/1.73m$^2$ | 0.98 | 0.96-0.99 | < 0.0001 |

RR: relative risk; CI: confidence interval; eGFR: estimated glomerular filtration rate

**Supplementary Table 5. Univariate and multivariate Cox regression analysis of risk factors at baseline for cardiovascular mortality**

| **miR-223** | | | |
|---|---|---|---|
| Models of patient survival (events = 29) | **RR** | **95% CI** | **p** |
| *Unadjusted* | | | |
| ln miR-223 | 0.75 | 0.49-1.12 | 0.162 |
| *Model adjusted for age, sex, and diabetes* | | | |
| ln miR-223 | 0.85 | 0.50-1.19 | 0.482 |
| Age per 1 year | 1.07 | 1.03-1.11 | <0.0001 |
| Male | 1.83 | 0.81-4.13 | 0.147 |
| Diabetes | 2.78 | 1.33-5.78 | 0.006 |
| *Model adjusted for age, sex, diabetes, and baseline eGFR* | | | |
| ln miR-223 | 0.89 | 0.54-1.45 | 0.633 |
| Age per 1 year | 1.07 | 1.03-1.12 | 0.001 |
| Male | 2.11 | 0.89-5.00 | 0.091 |
| Diabetes | 3.25 | 1.50-7.07 | 0.003 |
| Baseline eGFR per 1 ml/min/1.73m$^2$ | 1.00 | 0.98-1.02 | 0.983 |

RR: relative risk; CI: confidence interval; eGFR: estimated glomerular filtration rate.

| **miR-126** | | | |
|---|---|---|---|
| Models of patient survival (events = 29) | **RR** | **95% CI** | **p** |
| *Unadjusted* | | | |
| **ln miR-126** | **0.54** | **0.34-0.87** | **0.012** |
| *Model adjusted for age, sex, and diabetes* | | | |
| ln miR-126 | 0.66 | 0.40-1.11 | 0.666 |
| Age per 1 year | 1.07 | 1.03-1.11 | 0.001 |
| Male | 1.83 | 0.81-4.12 | 0.146 |
| Diabetes | 2.79 | 1.34-5.81 | 0.006 |
| *Model adjusted for age, sex, diabetes, and baseline eGFR* | | | |
| ln miR-126 | 0.69 | 0.41-1.18 | 0.177 |
| Age per 1 year | 1.07 | 1.03-1.12 | 0.001 |
| Male | 2.09 | 0.88-4.94 | 0.093 |
| Diabetes | 3.23 | 1.48-7.02 | 0.003 |
| Baseline eGFR per 1 ml/min/1.73m$^2$ | 1.00 | 0.98-1.02 | 0.919 |

RR: relative risk; CI: confidence interval; eGFR: estimated glomerular filtration rate

**Supplementary Table 6. Univariate and multivariate Cox regression analyses of risk factors at baseline for cardiovascular events**

| miR-223 | | | |
|---|---|---|---|
| Models of patient survival (events = 173) | **RR** | **95% CI** | **p** |
| *Unadjusted* | | | |
| **ln miR-223** | **0.69** | **0.58-0.81** | **< 0.0001** |
| *Model adjusted for age, sex, and diabetes* | | | |
| **ln miR-223** | **0.76** | **0.76-0.98** | **0.004** |
| Age per 1 year | 1.04 | 1.03-1.06 | < 0.0001 |
| Male | 1.54 | 1.11-2.14 | 0.009 |
| Diabetes | 1.87 | 1.34-2.53 | < 0.0001 |
| *Model adjusted for age, sex, diabetes, and baseline eGFR* | | | |
| ln miR-223 | 0.87 | 0.71-1.06 | 0.176 |
| Age per 1 year | 1.04 | 1.02-1.05 | < 0.0001 |
| Male | 1.64 | 1.16-2.31 | 0.005 |
| Diabetes | 2.00 | 1.46-2.76 | < 0.0001 |
| Baseline eGFR per 1 ml/min/1.73m$^2$ | 0.98 | 0.97-0.99 | 0.012 |

RR: relative risk; CI: confidence interval; eGFR: estimated glomerular filtration rate.

| miR-126 | | | |
|---|---|---|---|
| Models of patient survival (events = 173) | **RR** | **95% CI** | **p** |
| *Unadjusted* | | | |
| **ln miR-126** | **0.64** | **0.52-0.79** | **< 0.0001** |
| *Model adjusted for age, sex, and diabetes* | | | |
| **ln miR-126** | **0.76** | **0.61-0.96** | **0.019** |
| Age per 1 year | 1.04 | 1.03-1.06 | < 0.0001 |
| Male | 1.60 | 1.16-2.18 | 0.004 |
| Diabetes | 1.87 | 1.38-2.54 | < 0.0001 |
| *Model adjusted for age, sex, diabetes, and baseline eGFR* | | | |
| ln miR-126 | 0.86 | 0.71-1.06 | 0.176 |
| Age per 1 year | 1.03 | 1.02-1.05 | <0.0001 |
| Male | 1.67 | 1.19-2.35 | 0.003 |
| Diabetes | 1.99 | 1.45-2.75 | <0.0001 |
| Baseline eGFR per 1 ml/min/1.73m$^2$ | 0.98 | 0.98-0.99 | 0.007 |

RR: relative risk; CI: confidence interval; eGFR: estimated glomerular filtration rate.

**Supplementary Table 7. Univariate and multivariate Cox regression analysis of risk factors at baseline for atheromatous cardiovascular events.**

| miR-223 | | | |
|---|---|---|---|
| Models of patient survival (events = 119) | **RR** | **95% CI** | **p** |
| *Unadjusted* | | | |
| **ln miR-223** | **0.77** | **0.63-0.95** | **0.015** |
| *Model adjusted for age, sex, and diabetes* | | | |
| ln miR-223 | 0.87 | 0.69-1.08 | 0.209 |
| Age per 1 year | 1.04 | 1.02-1.05 | <0.0001 |
| Male | 1.63 | 1.10-2.42 | 0.015 |
| Diabetes | 2.16 | 1.50-3.12 | <0.0001 |
| *Model adjusted for age, sex, diabetes, and baseline eGFR* | | | |
| ln miR-223 | 0.97 | 0.78-1.27 | 0.834 |
| Age per 1 year | 1.03 | 1.01-1.05 | <0.0001 |
| Male | 1.72 | 1.14-2.60 | 0.010 |
| Diabetes | 2.16 | 1.47-3.17 | <0.0001 |
| Baseline eGFR per 1 ml/min/1.73m$^2$ | 0.99 | 0.98-0.99 | 0.025 |

RR: relative risk; CI: confidence interval; eGFR: estimated glomerular filtration rate.

| miR-126 | | | |
|---|---|---|---|
| Models of patient survival (events = 119) | **RR** | **95% CI** | **p** |
| *Unadjusted* | | | |
| ln miR-126 | 0.80 | 0.61-1.05 | 0.103 |
| *Model adjusted for age, sex, and diabetes* | | | |
| ln miR-126 | 0.95 | 0.71-1.28 | 0.755 |
| Age per 1 year | 1.04 | 1.02-1.05 | <0.0001 |
| Male | 1.67 | 1.13-2.47 | 0.010 |
| Diabetes | 2.17 | 1.50-3.13 | <0.0001 |
| *Model adjusted for age, sex, diabetes, and baseline eGFR* | | | |
| ln miR-126 | 1.08 | 0.78-1.49 | 0.639 |
| Age per 1 year | 1.03 | 1.01-1.05 | <0.0001 |
| Male | 1.73 | 1.15-2.61 | 0.019 |
| Diabetes | 2.16 | 1.47-3.17 | <0.0001 |
| Baseline eGFR per 1 ml/min/1.73m$^2$ | 0.98 | 0.98-0.99 | 0.017 |

RR: relative risk; CI: confidence interval; eGFR: estimated glomerular filtration rate.

**Supplementary Table 8. Univariate and multivariate Cox regression analysis of risk factors at baseline for non-atheromatous cardiovascular events.**

| **miR-223** | | | |
|---|---|---|---|
| Models of patient survival (events = 71) | **RR** | **95% CI** | **p** |
| *Unadjusted* | | | |
| **ln miR-223** | **0.61** | **0.46-0.78** | **<0.0001** |
| *Model adjusted for age, sex, and diabetes* | | | |
| **ln miR-223** | **0.65** | **0.48-0.88** | **0.004** |
| Age per 1 year | 1.04 | 1.03-1.07 | <0.0001 |
| Male | 1.14 | 0.70-1.87 | 0.582 |
| Diabetes | 2.08 | 1.22-3.12 | 0.005 |
| *Model adjusted for age, sex, diabetes, and baseline eGFR* | | | |
| ln miR-223 | 0.78 | 0.57-1.08 | 0.134 |
| Age per 1 year | 1.03 | 1.01-1.05 | 0.001 |
| Male | 1.63 | 0.70-1.98 | 0.511 |
| Diabetes | 2.32 | 1.40-3.82 | 0.001 |
| Baseline eGFR per 1 ml/min/1.73m$^2$ | 0.98 | 0.98-1.00 | 0.091 |

RR: relative risk; CI: confidence interval; eGFR: estimated glomerular filtration rate.

| **miR-126** | | | |
|---|---|---|---|
| Models of patient survival (events = 71) | **RR** | **95% CI** | **p** |
| *Unadjusted* | | | |
| **ln miR-126** | **0.49** | **0.37-0.66** | **<0.0001** |
| *Model adjusted for age, sex, and diabetes* | | | |
| **ln miR-126** | **0.58** | **0.43-0.78** | **0.001** |
| Age per 1 year | 1.04 | 1.02-1.07 | <0.0001 |
| Male | 1.59 | 1.07-2.35 | 0.022 |
| Diabetes | 1.94 | 1.21-3.09 | 0.006 |
| *Model adjusted for age, sex, diabetes, and baseline eGFR* | | | |
| ln miR-126 | 0.65 | 0.46-1.02 | 0.120 |
| Age per 1 year | 1.03 | 1.01-1.05 | 0.002 |
| Male | 1.20 | 0.72-2.00 | 0.486 |
| Diabetes | 2.29 | 1.40-3.04 | 0.001 |
| Baseline eGFR per 1 ml/min/1.73m$^2$ | 0.98 | 0.98-0.99 | 0.073 |

RR: relative risk; CI: confidence interval; eGFR: estimated glomerular filtration rate.

**Supplementary Table 9. Univariate and multivariate Cox regression analyses of risk factors at baseline for renal events (dialysis or transplantation).**

| **miR-223** | | | |
|---|---|---|---|
| Models of patient survival (events = 57) | **RR** | **95% CI** | **p** |
| *Unadjusted* | | | |
| **ln miR-223** | **0.49** | **0.36-0.67** | **< 0.0001** |
| *Model adjusted for age, sex, and diabetes* | | | |
| **ln miR-223** | **0.46** | **0.33-0.63** | **< 0.0001** |
| Age per 1 year | 0.98 | 0.96-0.99 | 0.004 |
| Male | 1.77 | 0.99-3.13 | 0.051 |
| Diabetes | 2.85 | 1.63-4.99 | < 0.0001 |
| *Model adjusted for age, sex, diabetes, and baseline eGFR* | | | |
| ln miR-223 | 0.91 | 0.66-1.23 | 0.905 |
| Age per 1 year | 0.95 | 0.93-0.97 | < 0.0001 |
| Male | 2.04 | 1.14-3.64 | 0.016 |
| Diabetes | 2.32 | 1.32-4.08 | 0.003 |
| Baseline eGFR per 1 ml/min/1.73m$^2$ | 0.89 | 0.86-0.91 | < 0.0001 |

RR: relative risk; CI: confidence interval; eGFR: estimated glomerular filtration rate.

| **miR-126** | | | |
|---|---|---|---|
| Models of patient survival (events = 57) | **RR** | **95% CI** | **p** |
| *Unadjusted* | | | |
| **ln miR-126** | **0.66** | **0.46-0.95** | **0.024** |
| *Model adjusted for age, sex, and diabetes* | | | |
| **ln miR-126** | **0.62** | **0.44-0.88** | **0.008** |
| Age per 1 year | 0.98 | 0.96-0.99 | 0.009 |
| Male | 2.01 | 1.13-3.57 | 0.017 |
| Diabetes | 2.78 | 1.59-4.88 | < 0.0001 |
| *Model adjusted for age, sex, diabetes, and baseline eGFR* | | | |
| ln miR-126 | 0.81 | 0.53-1.23 | 0.320 |
| Age per 1 year | 0.95 | 0.93-0.96 | < 0.0001 |
| Male | 2.16 | 1.21-3.84 | 0.009 |
| Diabetes | 2.33 | 1.32-4.10 | 0.003 |
| Baseline eGFR per 1 ml/min/1.73m$^2$ | 0.88 | 0.86-0.91 | < 0.0001 |

RR: relative risk; CI: confidence interval; eGFR: estimated glomerular filtration rate
